# Foot-Ball Casualties

**Published:** 1894-06

**Authors:** 


### FOOT-BALL CASUALTIES.

Lovers of foot-ball will find some very cheerful reading in a late article in the New York *Medical Examiner*, entitled the "Foot-Ball Butcher's Bill." It recites the casualties that have occurred in the foot-ball field in England within the last two years. In the season of 1892–93 there were thirty-one deaths. Thirty-nine legs were broken, twenty-five collar bones and twelve arms, besides seventy-five miscellaneous injuries. The season just closed has furnished twenty-one deaths, seventy-nine broken legs, seventy-two broken collar-bones and twenty-four broken arms. The miscellaneous injuries include an interesting list of brain concussions, dislocated joints, smashed ribs, broken fingers, disarranged noses and endless contusions. Two of the deaths last season occurred "among the spectators who died from over-excitement and exertion in cheering their favorite teams during the progress of a match."

In the United States last season there were eight deaths from the game and one hundred and thirty-six young men seriously injured by fractures, dislocations, concussions and the like.

The modern game is nothing short of a wholesale fight, severe enough, it seems, to cause death even in an on-looker. Both the barbarities of the game and the enthusiasm of the spectators should be toned down to a point consistent with safety to life.

---

THE last International Medical Congress at Rome (March 28th to April 5th) is said to have been the most successful ever held. The Congress was formally opened by the King and Queen of Italy, and Crispi, Prime Minister, welcomed the foreign delegates.

More than seven thousand medical men were registered. The meetings were held in the large polyclinic building under the Presidency of Guido Bacelli. President Bacelli is a professor in the Roman University and Minister of Public Instruction in the Cabinet of King Humbert.

More than 3,000 papers were handed in. About 200 Americans were in attendance upon the Congress. Dr. Murphy, of Chicago, read a paper on "Cholecystenterostomy," and reported twenty cases successfully treated by the Murphy button. Dr. Manley, of New York, produced a good paper on "Spinal Surgery." Dr. Edebohls, of New York, described a new method of shortening the round ligaments. Dr. Stewart, of New York, read a good paper in the obstetrical section on "Urinary Testing in Puerperal Fever."

Among other papers read were: "Management of Typhoid Fever," by Burney Yeo, of London; "The Treatment of Certain Neuroses by Cerebrine," by Althaus, of London ; "The Parasitism of Cancer," by Foa, of Turin; "The Treatment of Peritonitis," by Tait, of Birmingham. Championnierre, of Paris, read a paper on a "Clinical Study of Sixty-four Cases of Trephining." One entire day in the obstetrical section was devoted to a discussion of Symphyseotomy. Bastianelli, of Rome, described his new method of making a gastro-enterostomy by means of the Paquelin cautery, which created much interest.

Neisser read an exhaustive paper on "The Gonococcus and its relation to Blennorrhea." "The Treatment of Syphilis" was discussed by Schiff, of Vienna, who advised excision of the initial lesion.

The correspondent of the *Times and Register* says that the Roman police seem to think that all Americans live in Chicago, and when an Englishman or one from the States asks one of them a question he shrugs his shoulders and points to an office where there is an interpreter and says, "he spik shecarg."

The next meeting of the Congress will be held in Russia at a time and place to be determined by the President.

